# Physiological and stoichiometric characterization of ethanol-based chain elongation in the absence of short-chain carboxylic acids

**DOI:** 10.1038/s41598-023-43682-x

**Published:** 2023-10-13

**Authors:** Maximilienne Toetie Allaart, Bartholomeus B. Fox, Ingo H. M. S. Nettersheim, Martin Pabst, Diana Z. Sousa, Robbert Kleerebezem

**Affiliations:** 1 Department of Biotechnology, Delft University of Technology, Delft, The Netherlands; 2 Laboratory of Microbiology, Wageningen University & Research, Wageningen, The Netherlands

**Keywords:** Applied microbiology, Bacteria, Microbial communities, Environmental microbiology, Environmental biotechnology, Industrial microbiology

## Abstract

Hexanoate is a valuable chemical that can be produced by microorganisms that convert short-chain- to medium-chain carboxylic acids through a process called chain elongation. These microorganisms usually produce mixtures of butyrate and hexanoate from ethanol and acetate, but direct conversion of ethanol to hexanoate is theoretically possible. Steering microbial communities to ethanol-only elongation to hexanoate circumvents the need for acetate addition and simplifies product separation. The biological feasibility of ethanol elongation to hexanoate was validated in batch bioreactor experiments with a *Clostridium kluyveri*-dominated enrichment culture incubated with ethanol, acetate and butyrate in different ratios. Frequent liquid sampling combined with high-resolution off-gas measurements allowed to monitor metabolic behavior. In experiments with an initial ethanol-to-acetate ratio of 6:1, acetate depletion occurred after ± 35 h of fermentation, which triggered a metabolic shift to direct conversion of ethanol to hexanoate despite the availability of butyrate (± 40 mCmol L^−1^). When only ethanol and no external electron acceptor was supplied, stable ethanol to hexanoate conversion could be maintained until 60–90 mCmol L^−1^ of hexanoate was produced. After this, transient production of either acetate and butyrate or butyrate and hexanoate was observed, requiring a putative reversal of the Rnf complex. This was not observed before acetate depletion or in presence of low concentrations (40–60 mCmol L^−1^) of butyrate, suggesting a stabilizing or regulatory role of butyrate or butyrate-related catabolic intermediates. This study sheds light on previously unknown versatility of chain elongating microbes and provides new avenues for optimizing (waste) bioconversion for hexanoate production.

## Introduction

Microbial chain elongation is the anaerobic conversion of short-chain carboxylic acids (SCCAs) to medium-chain carboxylic acids (MCCAs) using ethanol or lactate as electron donor^1–3^. Chain elongation has gained momentum over the past two decades, as it can be used to convert a broad range of (organic) waste streams to biochemical building blocks like *n-*butyrate (butyrate), *n-*hexanoate (hexanoate) and *n-*octanoate (octanoate)^4–8^. Especially hexanoate has gained significant interest as a product of microbial chain elongation, both for its properties as antimicrobial agent, food and feed additive and biofuel precursor, and for its market value^9,10^.

Microbial communities are good catalysts for waste-to-value conversions. They allow digestion of complex waste streams through complementary functionalities in the microbiome and resilience to changes in operational parameters (temperature, influent composition etc.) due to functional redundancies. Furthermore, high costs for maintaining aseptic environments are omitted^9,11^. Therefore, improving the understanding of the mechanisms that drive the physiology of chain elongating communities is important for industrial process development^12^.

In the case of chain elongation with ethanol as electron donor, the thermodynamically unfavorable ethanol oxidation (EO, Eq. 1) is coupled to reversed β-oxidation (RBO) of a SCCA Eq. 2—acetate). Furthermore, electron bifurcation in RBO leads to the production of reduced ferredoxin, which is required for hydrogen formation^13,14^. To enable the production of two H_2_ from EO, at least two RBO cycles should run to provide sufficient reduced ferredoxin. Experimentally, however, EO and RBO are often observed to be coupled in a 1:5 ratio (Eqs. 3 and 4)^15,16^. This facilitates the production of more reduced ferredoxin, which is used by the proton translocating Rnf complex to build up a chemiosmotic gradient. Additional ATP can be harvested from this gradient. Cellular redox balance can theoretically also be maintained when RBO operates independently of EO, rendering flexibility in their actual stoichiometric coupling^17^.

1 $$ \begin{gathered} Ethanol \to Acetate + H^{ + } + 2 H_{2} \hfill \\ \Delta G^{01} = + 41.5 {\text{kJ/mol}} \hfill \\ \end{gathered} $$

2 $$ \begin{gathered} Ethanol + Acetate \to Butyrate \hfill \\ \Delta G^{01} = - 38.7 {\text{kJ/mol}} \hfill \\ \end{gathered} $$

3 $$ \begin{gathered} 6 Ethanol + 4 Acetate \to 5 Butyrate + H^{ + } + 2 H_{2} + H_{2} O \hfill \\ \Delta G^{01} = - 183.6 {\text{kJ/mol}} \hfill \\ \end{gathered} $$

4 $$ \begin{gathered} 6 Ethanol + 5 Butyrate \to 5 Hexanoate + Acetate + H^{ + } + 2 H_{2} + H_{2} O \hfill \\ \Delta G^{01} = - 184.6 {\text{kJ/mol}} \hfill \\ \end{gathered} $$

Even though the stoichiometric coupling of EO and RBO is flexible, few studies quantify the employed stoichiometry of chain elongators. Instead, a focus on the treatment of specific waste streams, process intensification or product spectrum control is distinguished in literature on chain elongation^17–22^.

**Figure 1:**
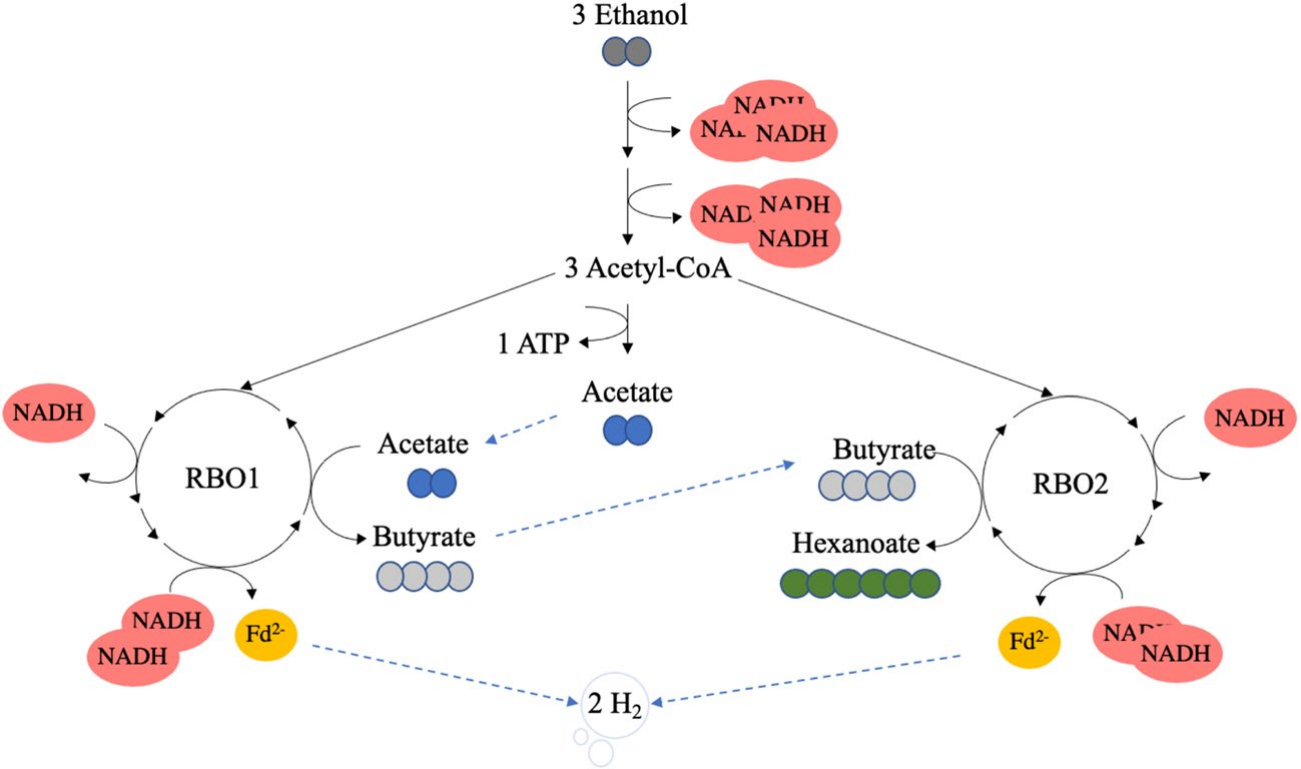
Schematic representation of the pathway for chain elongation of ethanol to hexanoate in the absence of external electron acceptor. In this stoichiometry ethanol oxidation and reversed β-oxidation are coupled in a 1:2 ratio, and acetate is subsequently elongated to butyrate and hexanoate. Energy conservation only happens via substrate-level phosphorylation, not via harvesting of proton motive force generated via the Rnf complex.

Understanding the conditions that trigger different stoichiometric couplings between EO and RBO enables product spectrum control. For example, the elongation of ethanol to hexanoate without external electron acceptor is biochemically balanced (Eq. 5, Fig. 1)^17,23^. Interestingly, conversion of ethanol to hexanoate in the absence of acetate and butyrate has been observed by Smith et al.^24^. However, other authors reported an absolute requirement of acetate for the elongation of ethanol to butyrate and hexanoate^25,26^. The discrepancy between these results makes it unclear whether and in which conditions ethanol can be converted to hexanoate in absence of acetate or butyrate as electron acceptor:

5 $$ \begin{gathered} 3 Ethanol \to Hexanoate + H^{ + } + 2 H_{2} + H_{2} O \hfill \\ \Delta G^{01} = - 67.9 {\text{kJ/mol}} \hfill \\ \end{gathered} $$

We theorize that the elongation of ethanol to hexanoate is the metabolic extreme of chain elongating catabolism. This conversion encompasses a 1:2 stoichiometric ratio between EO and RBO and elongates internally generated acetate and butyrate subsequently to hexanoate. This can theoretically happen even in the complete absence of external electron accepting SCCAs. We therefore hypothesize that this stoichiometry will be exploited under electron acceptor limitation. In this work, we investigate the effect of omitting electron accepting SCCAs (acetate and butyrate) on the physiology and stoichiometry of chain elongating microorganisms. Inocula were taken from an active anaerobic sequencing batch bioreactor (ASBR) performing ethanol-based chain elongation Allaart et al.^27^ grown in medium with only ethanol, medium with ethanol and acetate in a 6:1 ratio and medium with ethanol and butyrate in a 20:1 ratio. In the ethanol-only experiment the proteome was analyzed at time points where metabolic switching occurred.

## Materials and methods

### Batch chain elongation experiments

**Figure 2:**
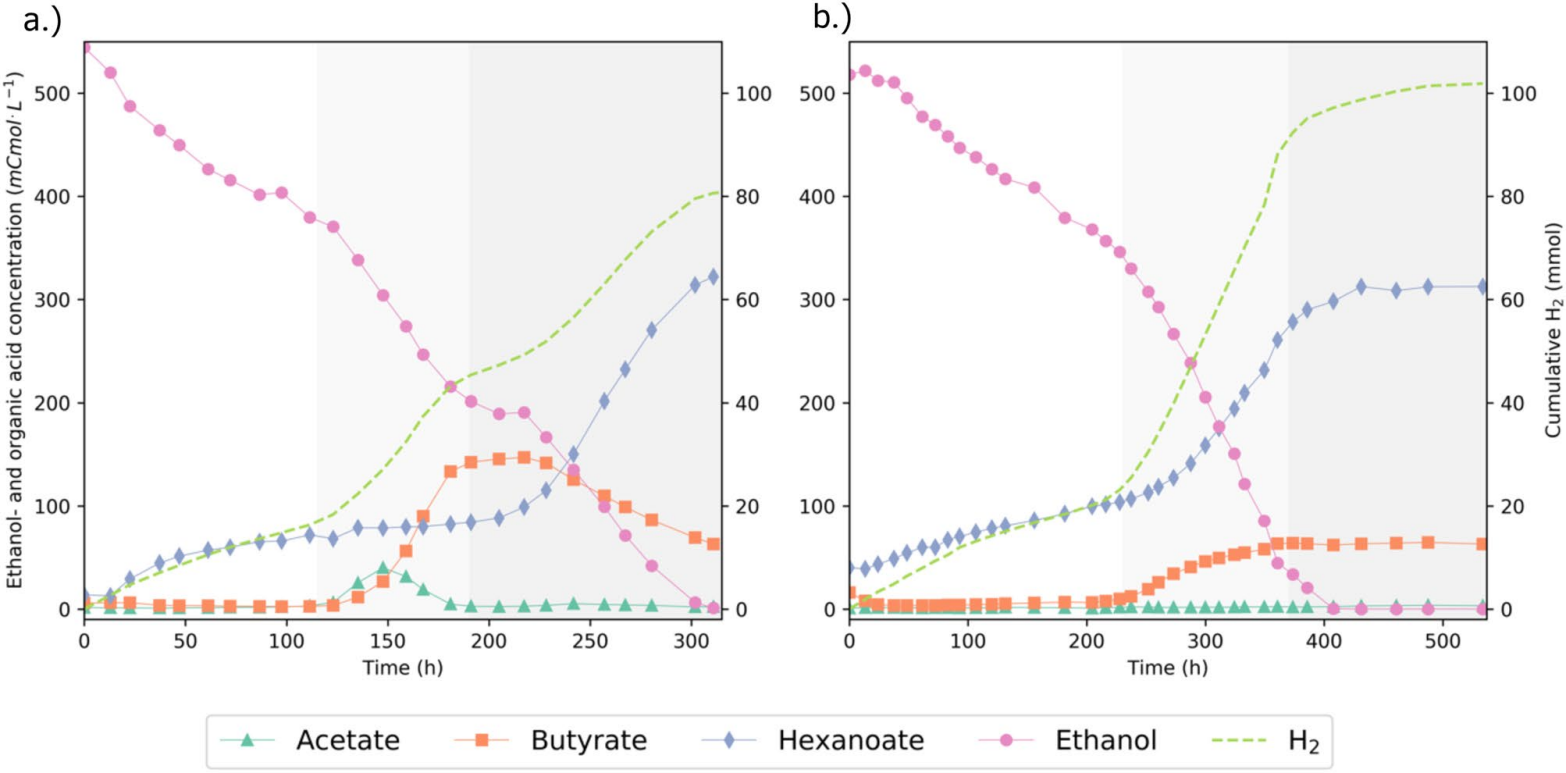
Ethanol-only batch experiment replicate I (**A**) and II (**B**). The replicates show different biochemical conversion profiles of ethanol. 100 mL of an active chain-elongating culture was incubated in a batch bioreactor with 500 mCmol ethanol and no added SCCAs in duplicate. Different phases in the experiment are distinguished according to stoichiometric shifts and are indicated with different shades of background color. Solid lines are linear interpolations between the datapoints.

**Figure 3:**
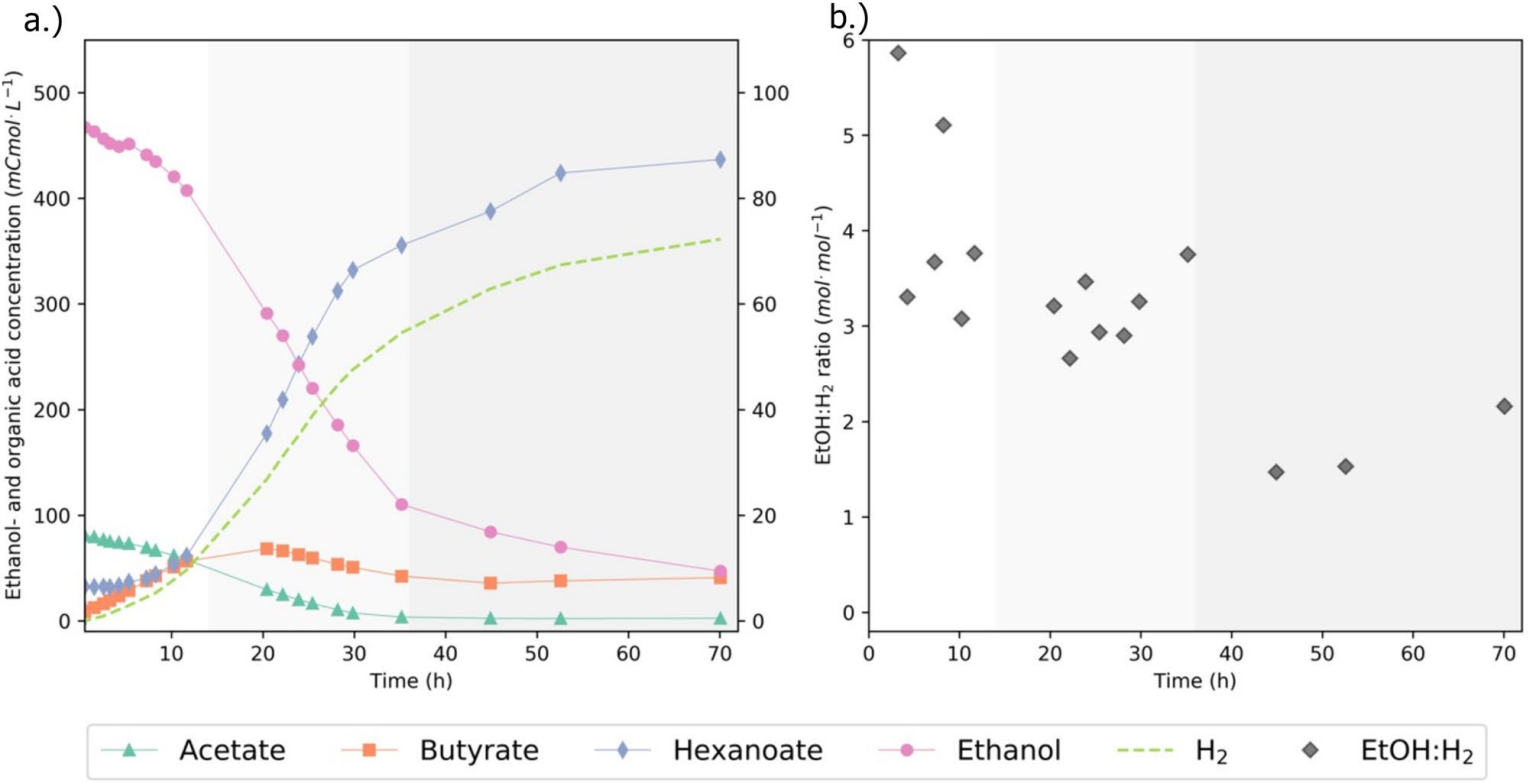
Biochemical conversion profiles (**A**) and EtOH:H_2_ ratios (**B**) during ethanol chain elongation in presence of acetate. 100 mL of an active chain-elongating culture was incubated in a batch bioreactor with 484 mCmol ethanol and 82 mCmol acetate, leading to an ethanol:acetate ratio of 6:1 at the start of the experiment. Different phases in the experiment are distinguished with different shades of background color. EtOH:H_2_ ratios were calculated using the point-wise difference between consumed ethanol and produced hydrogen, outliers due to unreliable ethanol measurements were removed. Solid lines are linear interpolations between the data points.

**Figure 4:**
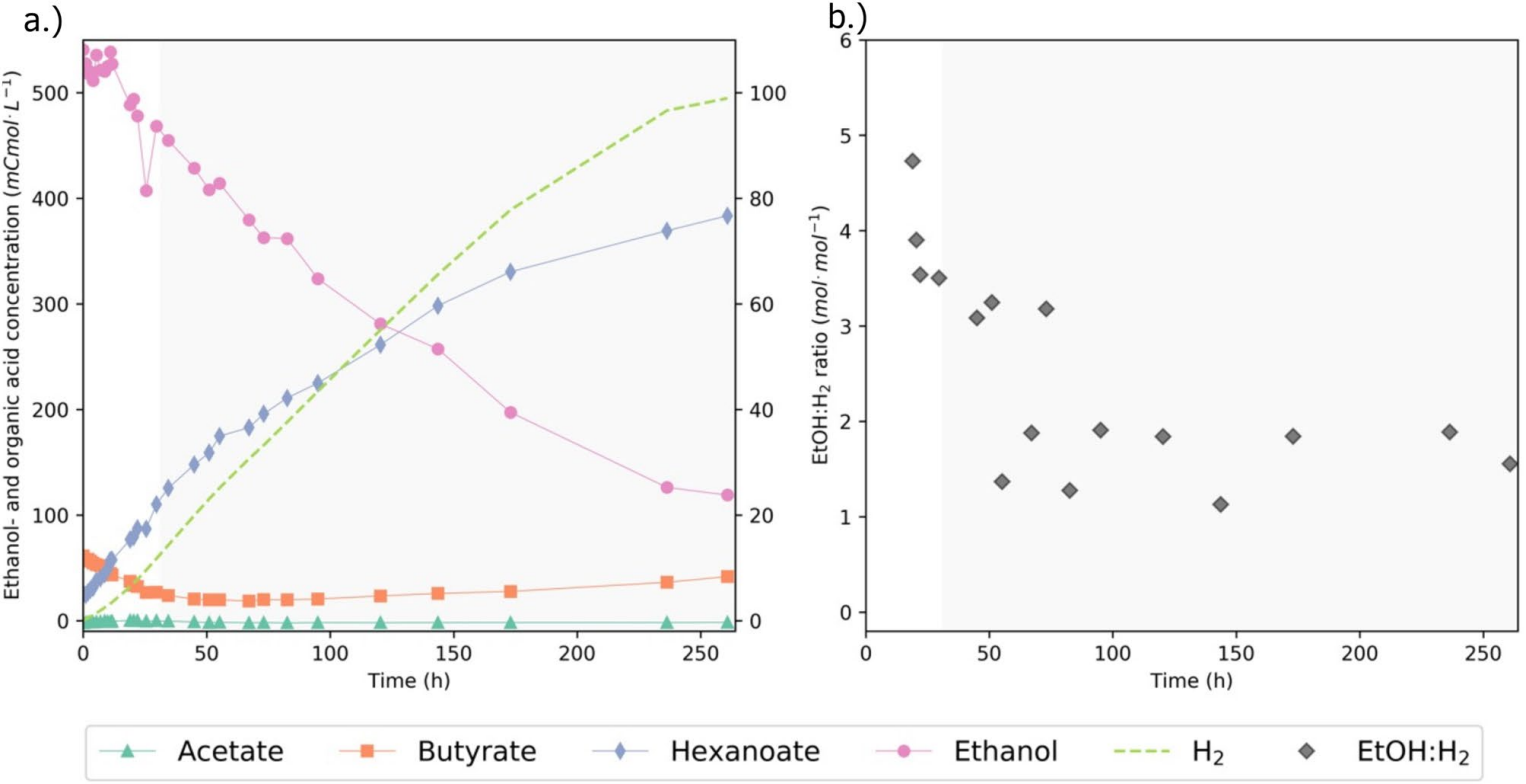
Biochemical conversion profiles (**A**) and EtOH:H_2_ ratios (**B**) during ethanol chain elongation in presence of butyrate. 100 mL of an active chain-elongating culture was incubated in a batch bioreactor with 550 mCmol ethanol and 60 mCmol butyrate, leading to an ethanol:butyrate molar ratio of 19:1 at the start of the experiment. Different phases in the experiments are distinguished according to stoichiometric shifts and are indicated with different shades of background color. EtOH:H_2_ ratios were calculated using the point-wise difference between consumed ethanol and produced hydrogen. Due to fluctuating ethanol measurements in the first 21 h, the EtOH:H_2_ ratio in this phase was calculated from t = 0 to t = 19 and from t = 0 to t = 20.5 with the initial concentration of ethanol as reference. Solid lines are linear interpolations between the data points.

Batch experiments were carried out at pH 7.0 ± 0.2 in in a 2L non-sterile bioreactor with an initial working volume of 1L (Applikon, Delft, The Netherlands). The reactor was continuously agitated at a speed of 400 rpm (SC4, DASGIP, Germany) using mechanical stirrers. The temperature was maintained at 34 °C using a water jacket and a thermostat bath (E300, Lauda, Germany). Anaerobic conditions were ensured by continuously sparging the reactors with 50 ± 5 mL min^-1^ (MX44, DASGIP, Germany) of N_2_/CO_2_ (95:5vol%). To prevent culture broth evaporation, the off-gas was cooled using a cryostat set to 5 °C. The amounts of substrates added to the bioreactor in each experiment are summarized in Table 1. The batch experiments were inoculated with 100 mL of a culture retrieved from an ASBR enrichment^27^. This culture was highly enriched in *Clostridium kluyveri* and contained approximately 10 mmol ethanol, 0.6 mmol acetate, 2.0 mmol butyrate and 4.0 mmol hexanoate. 90 g of 10 × concentrated nutrient stock solution was added to each batch experiment to enable exponential growth. To ensure anaerobic conditions before initiation of the batch experiment, the carbon source and inoculum were added when no more oxygen was measured in the off-gas. A biological replicate was performed for all three conditions. The replicate of experiment 2 was performed with a reduced sampling frequency to confirm that sampling has a negligible effect on the biological conversions. Only 5 samples of 3 mL were taken from this system during the operating time of ± 120 h, whereas 21 samples of 4 mL were taken from the regularly sampled replicate. The raw data presented in Figs. 2, 3 and 4 can be found in Supplementary Table S1.

**Table 1:** Added amounts of susbtrate in batch chain elongation experiments.

Experiment no.	Acetate (mmol)	Ethanol (mmol)	Butyrate (mmol)	Hexanoate (mmol)
1	0	250	0	0
2	41	242	0	0
3	0	275	15	0

### Cultivation medium

The nutrients stock contained 67.6 mM KH_2_PO_4_, 93.5 mM NH_4_Cl, 4.1 mM MgSO_4_·7 H_2_O, 5.9 mM MgCl_2_·6 H_2_O, 50 mM 2-bromoethanesulfonate (BES) (TCI, Tokyo, Japan), 20 mL of alkaline trace elements solution, 20 mL of SL-10 trace elements solution and 20 mL of B vitamin solution. The alkaline trace elements solution contained (in g L^−1^): NaOH 0.4, Na_2_SeO3 0.017, Na_2_WO_4_·2 H_2_O 1.03, Na_2_MoO_4_.2H2O 0.024. The SL-10 trace elements solution contained (in g L^−1^): FeCl_2_·4 H_2_O 1.5, FeCl_3_·6 H_2_O 2.5, ZnCl_2_ 0.07, MnCl_2_·4 H_2_O 0.1, H_3_BO_3_ 0.006, CoCl_2_·6 H_2_O 0.19, CuCl_2_·2 H_2_O 0.002, NiCl_2_ ·6 H_2_O 0.024, Na_2_MoO_4_ ·2 H_2_O 0.036 and 10 mL 25% HCl. The B vitamin solution contained (in g L^-1^): biotin 0.02, nicotinamide 0.2, p-aminobenzoic acid 0.1, thiamine hydrochloride 0.2, Ca-pantothenate 0.1, pyridoxamine 0.5, cyanocobalamine 0.1, riboflavin 0.1.

### Analytical methods

The off-gas composition (N_2_, CO_2_, H_2_, O_2_, CH_4_) of the reactors was measured using mass spectrometry (PRIMA BT Benchtop, Thermo Scientific, UK). Acid- and base dosage were monitored using an integrated revolution counter (MP8, DASGIP, Germany). Biomass concentrations (Supplementary Figure S4) were monitored both by measuring optical density at 660 nm (OD_660_) and the amount of volatile suspended solids (VSS) using 150 mL culture broth^28^, calculated assuming a biomass composition of CH_1.8_O_0.5_N_0.2_ and molecular weight of 24.6 g Cmol^−1^. After re-start of the enrichment, the cell pellets were washed with PBS before drying as the remaining supernatant after centrifuging interfered with correct biomass determination. The biomass measurements of the first batch experiment were corrected using a spectrophotometric Total Organic Nitrogen measurement (LCK 138, Hach-Lange, Germany) with frozen stored pellets from the enrichment reactor at the date of batch inoculation. Technical duplicates of OD, VSS and TON measurements were always performed. Ethanol, acetate, butyrate and hexanoate concentrations were determined using either high performance liquid chromatography (HPLC) or gas chromatography (GC). HPLC was performed using an Aminex HPX-87H column (BioRad, USA) at 59 °C coupled to an ultraviolet detector at 210 nm (Waters, USA) with 1.5 mmol L^−1^ phosphoric acid as eluent. Biomass was removed from the reactor samples by centrifugation and filtration using a 0.22 µm membrane filter (Millipore, Millex-GV, Ireland). GC was performed using a Trace 1300 machine (Thermo Scientific, USA) equipped with an injector maintained at 180 °C and a carbowax polyethylene glycol column of 20 m × 0.18 mm (Agilent, USA). A temperature gradient was used from 50 to 180 °C over 24 min. Helium was used as carrier gas and fermentation substrates and products were detected using a flame ionization detector set at 200 °C. Iso-hexanoic acid was used as internal standard and samples were acidified using pure formic acid (Sigma Aldrich, US).

### Microbial community analysis

The microbial community composition in the enrichment reactor was analyzed using amplicon sequencing of the 16S rRNA gene as described in^27^ to confirm culture stability over time. The sequences have been stored in the 4TU research database and can be found under the https://doi.org/10.4121/f74b503a-81d5-4187-b696-ba9c8133d2a3. Furthermore, de novo composition analysis was performed from the proteome data at two different timepoints in the enrichment.

### Proteomics sample preparation

Protein extraction from chain elongating microbial community pellets (250 µL cell pellet slurry of pellets from 50 mL culture broth) was performed in solution (protocol adapted from^29^. In short, the pellet slurries were resuspended in 250 µL 50 mM triethylammonium bicarbonate (TEAB) (Merck Sigma, Cat. No. T7408) containing 1% (w/w) NaDOC and 250 µL B-PER™ Bacterial Protein Extraction Reagent (Thermo Fisher, cat# 78243). Cells were lysed by glass bead beating and vortexing 6 times for 1.5 min alternated with 0.5 min rest on ice. The samples were kept in an Eppendorf ThermoMixer for 3 min at 80 °C and 1000 rpm and placed in an ultrasonic bath for 10 min for protein extraction. The proteins were precipitated by adding Trichloroacetic (TCA) solution 6.1N (Sigma Aldrich, T0699) in a 1:4 ratio to the supernatant and vortexing. Samples were incubated for 30 min at 4 °C and spun down for 15 min at 14.000 rcf for precipitation. The protein pellet was washed with ice-cold acetone and resuspended in 6 M Urea (cat# U5128, Sigma-Aldrich) in 200 mM ammonium bicarbonate. The extracted proteins were reduced by addition of 10 mM DTT (Merck Sigma, Cat. No. 43815) and incubated for 1 h at 37 °C and 300 rpm in an Eppendorf ThermoMixer. Subsequently, the proteins were alkylated for 30 min at room temperature in the dark by addition of 20 mM iodoacetamide (Merck Sigma, I1149). Proteolytic digestion was performed using Sequencing Grade Trypsin (Promega, Cat. No. V5111), 1:100 enzyme to protein ratio (v/v) and incubated at 37 °C and 300 rpm overnight. Solid phase extraction was performed with an Oasis HLB 96-well μElution plate (Waters, Milford, USA, Cat. No. 186001828BA). Eluates were dried using a SpeedVac vacuum concentrator. Dried peptides were resuspended in 3% ACN / 0.01% TFA prior to MS-analysis.

### Whole cell lysate shotgun proteomics

An aliquot of approx. 250 ng protein digest per sample was analysed in triplicates using a one-dimensional shotgun proteomics approach^30^. Briefly, the samples were analysed using a nano-liquid-chromatography system consisting of an EASY nano-LC 1200 equipped with an Acclaim PepMap RSLC RP C18 separation column (50 µm × 150 mm, 2 µm), and an QE plus Orbitrap mass spectrometer (Thermo Fisher Scientific, Germany). The flow rate was maintained at 350 nL/min over a linear gradient from 5 to 25% solvent B over 88 min, and finally to 55% B over 60 min. Data were acquired from 0 to 175 min. Solvent A was H_2_O containing 0.1% formic acid, and solvent B consisted of 80% acetonitrile in H_2_O and 0.1% formic acid. The Orbitrap was operating in data dependent acquisition mode measuring peptide signals form 385‒1250 m/z at 70 K resolution with a max IT of 75 ms and an AGC target of 3e6. The top 10 signals were isolated at a window of 2.0 m/z and fragmented using a NCE of 28. Fragments were acquired at 17 K resolution with a max IT of 75 ms and an AGC target of 2e5. Unassigned, singly and > 6 times charged mass peaks were excluded from fragmentation.

### Database searching

Mass spectrometric raw data were deposited in the PRIDE partner repository under accession number PXD040972 and first analysed using the NovoBridge pipeline^31^ to estimate the microbial composition of the enrichment. Protein reference sequences at the genus level covering the major taxa were further retrieved from UniprotKB to confirm the taxonomic (protein) composition and to investigate overall metabolic changes. A focused analysis was further performed using the reference proteome of the major taxon *C. kluyveri* (strain ATCC 8527/DSM 555/NCIMB 10,680). Database searching was performed using PEAKS X (Bioinformatics Solutions Inc., Canada) allowing for 20 ppm parent ion and 0.02 m/z fragment ion mass error, 3 missed cleavages, carbamidomethylation as fixed and methionine oxidation and N/Q deamidation as variable modifications. Peptide spectrum matches were filtered for 1% false discovery rate and protein identifications with ≥ 2 unique peptides were accepted as significant.

### Label free quantification

Label free quantification was performed using the PEAKS Q module (Bioinformatics Solutions Inc., Canada). A comparison of the protein expression level between conditions was performed on identified peptide spectra filtered for 1% false discovery rate, a mass error equal or less to 10 ppm and a maximum retention time shift between runs of 5 min. The significance of protein abundance changes within the groups (conditions) was determined using ANOVA. Considered proteins required at least 2 unique peptide identifications per protein sequence. The fold-change of protein levels obtained from PEAKSQ between the different samples was visualized with heatmaps using the seaborn package in Python 3.0.

### Carbon- and electron recovery and ethanol evaporation measurements

To confirm the validity of the measurements, carbon- and electron recoveries were calculated over the batch experiments. The bioreactor volume was corrected for sampling, considering a sample volume of 3 or 4 mL per sample, and base addition. Additionally, the amounts of substrates and products removed with sampling were accounted for. To test the significance of ethanol evaporation in the used setup, an abiotic test was carried out in the reactor system (Supplementary Fig. S2). The reactor contained 1L of a 250 mM ethanol solution and was sparged with 50 mL min^−1^ N_2_. Ethanol evaporation was quantified using liquid sampling and monitoring of ethanol in the off-gas using the MS signal at mass 15 (CH_3_^+^ fragment). The recovery of carbon and electrons was calculated by dividing the amount of C/e^-^ in the reactor, corrected for compound removal via sampling and via the off-gas, by the amount of C/e^-^ in the reactor at t0.

## Results

### Chain elongating microorganisms elongate ethanol in the absence of external electron acceptor

The capacity of chain elongating microorganisms to survive with ethanol as only carbon- and energy source was tested using a chain elongating culture highly enriched in *C. kluyveri.* The culture was inoculated in a batch bioreactor with mineral medium containing 500 mCmol ethanol and no additional SCCAs in duplicate. The transfer of residual SCCAs from the inoculum to the bioreactor led to an initial molar ethanol:acetate ratio of ± 416:1 and ethanol:butyrate ratio of ± 100:1. The resulting SCCA concentration was negligible compared to the available amount of ethanol (Fig. 2). The carbon- and electron balances in both replicates closed well at all sampled times after accounting for ethanol evaporation (Supplementary Figs. S7 and S8).

In the first phase of the experiment, ethanol was converted to hexanoate following the stoichiometry in Eq. (5) in both replicates. This conversion continued for approximately 100 and 200 h, respectively, after which 60–90 mC-M of hexanoate had accumulated. The conversion of ethanol to hexanoate could not be maintained until ethanol exhaustion. In both biological replicates a switch to a different, seemingly faster, catabolism was observed. This switch marked the start of the second phase of the experiment, shaded in light grey in Fig. 2. In replicate I the stoichiometric shift led to simultaneous accumulation of acetate, butyrate and hydrogen and a complete halt to hexanoate formation (Fig. 2A). In replicate II butyrate, hexanoate and hydrogen accumulated simultaneously (Fig. 2B). These stoichiometries occurred transiently after which the observed stoichiometry shifted again, marked by the dark grey shading in Fig. 2. In replicate I, canonical chain elongation of the accumulated acetate and butyrate occurred in the third phase of the experiment. This resulted in net hexanoate formation until all ethanol was consumed. In replicate II ethanol elongation to hexanoate was resumed until ethanol was exhausted. Overall, ethanol was consumed at a rate of ± 0.7–0.9 mmol h^-1^ when acetate and butyrate were not added to the medium.

### Community composition and proteome during ethanol-only chain elongation

To assess whether the observed physiologies correlated with shifts in community composition or relative protein expression levels, 16S rRNA gene amplicon sequencing and comparative metaproteomics were performed on replicate II of the ethanol-only batch experiment. Both methods indicated approx. 80–90% enrichment of the *Clostridium* genus and confirmed *C. kluyveri* as the major taxon (Supplementary Figure S5)*. Desulfovibrio* became more abundant during the experiment (up to approx. 15% based on protein biomass). Even though enzymes for sulfate reduction with ethanol or hydrogen as electron donor were detected, the net influence of sulfate reducing metabolisms on the overall stoichiometry was neglected due to minimal sulfate availability in the medium. Syntrophic ethanol oxidation and methane production were also excluded as no methane was measured in the off-gas in any of the replicates. Thus, the observed physiologies were all attributed to chain-elongating organisms. Therefore, the relative log2 fold-changes in protein levels in the biomass were mapped to the reference genome of the major taxon (*C. kluyveri)* to ensure high annotation accuracy.

The relative protein levels of central carbon metabolism proteins were analyzed at the start of phase II (t1) and III (t2) compared to the inoculum (t0) (Supplementary Fig. S6). Relative protein levels of all measured proteins can be found in Table S2. The proteome reflected that acetate kinase (ack) and cat3, which catalyzes the final transferase reaction of RBO, were more abundant in phases II and III. Increased abundance of acetate kinase indicates increased importance of ethanol oxidation to acetate in the applied conditions. Correspondingly, the levels of (several) alcohol dehydrogenases (aldh) and of phosphotransacetylase (pta) were also increased in phase II and III. Furthermore, increased amounts of the NADH dehydrogenase domain of the Rnf complex (RnfC) were distinguished (Supplementary Figure S6). However, due to the membrane-integral or -associated nature of the Rnf subunits, quantitative conclusions on Rnf levels could not be drawn^32^.

### Ethanol chain elongation in the presence of acetate

In this batch experiment, the influence of acetate supply on the stoichiometry and rate of chain elongation was assessed by supplying ethanol and acetate in a 6:1 molar ratio. Highly similar hydrogen profiles (Supplementary Fig. S1), fermentation times and final substrate- and product concentrations confirmed biological replicability and negligibility of sampling effects. Figure 3 shows the conversion profiles and EtOH:H_2_ ratios of the regularly sampled replicate. The carbon- and electron balances of this experiment closed well at all sampled timepoints (Supplementary Figs. S7 and S8). The initial phase of the experiment was characterized by exponential growth and exponentially increasing hydrogen production. In the second phase, growth became linear until acetate was depleted. Hexanoate production was favored over butyrate production during this phase. The ethanol consumption rate in the presence of acetate (phase I + II) was ± 5 mmol h^-1^. In the third phase of the experiment acetate was limiting. Butyrate did not get consumed completely and stayed constant at a concentration of 39.2 ± 2.8 mCmol L^-1^. Ethanol was consumed and hexanoate and hydrogen formation was observed. Furthermore, the EtOH:H_2_ ratio decreased substantially during this experimental phase to a value of 1.7 ± 0.4 (Fig. 3). The stoichiometry in Eq. (5) comprises an EtOH:H_2_ ratio of 1.5, and the ratio we measured was close to this theoretical value. This shows that the stoichiometry of Eq. (5) was likely the active catabolic stoichiometry under acetate limitation.

### Ethanol chain elongation in the presence of butyrate

To assess the effect of butyrate availability on ethanol metabolism, a third experiment was performed with a 19:1 initial ethanol:butyrate molar ratio. The added butyrate concentration was 60 mCmol L^-1^, which is in the order of magnitude of the concentrations at which butyrate consumption ceased in replicate II of the ethanol only batch and the ethanol + acetate batch. Figure 4 presents the data of replicate I of this experiment. The replicates of experiment 3 showed very comparable physiologies until 100 h of cultivation, after which no more hexanoate accumulated in replicate II (Supplementary Figure S2). The decrease in ethanol concentration that was still observed was most likely a result of evaporation (Supplementary Figure S3). After the experiment we discovered that the gas recirculation machine of replicate II was malfunctioning, which led to oxygen contamination in the reactor. Therefore, the data of replicate II is not directly included in our analysis. Figure 4 shows initial consumption of butyrate and formation of hexanoate. After this, a stoichiometric shift to ethanol elongation to hexanoate (indicated by light grey shading) was observed. The EtOH:H_2_ ratio during ethanol elongation to hexanoate was 1.9 ± 0.7 mol mol^−1^, which is slightly higher than the expected ratio of 1.5 (Fig. 1, Eq. 5). This could be attributed to an overestimation of the ethanol consumption due to evaporation of ethanol during the experiment (Supplementary Fig. S2). After ± 260 h of cultivation, ethanol was not fully depleted but the H_2_ fraction in the off-gas had dropped an order of magnitude. Therefore, it was assumed that there was no more metabolic activity and the experiment was terminated. The average ethanol consumption rate in the presence of small amounts of butyrate was ± 0.8 mmol h^−1^. This rate is comparable to the ethanol consumption rate in the absence of electron accepting SCCAs.

## Discussion

In this study, the effect of omitting electron accepting SCCAs (acetate and butyrate) on the physiology and stoichiometry of chain-elongating microorganisms was assessed. Our results show that chain-elongating microbial communities can grow under electron acceptor limitation by elongating ethanol to hexanoate, rendering a 1:2 coupling of EO:RBO. However, this stoichiometry could not be maintained until ethanol depletion and a switch to simultaneous production of an acetate-butyrate or butyrate-hexanoate mixture from ethanol was observed. Low concentrations (40–60 mCmol L^−1^) of butyrate prevented this transient physiology from occurring.

Various studies have shown that the supplied ratio of ethanol versus acetate strongly impacts the product spectrum of chain elongation^19,24,26,33,34^. Feeding more ethanol directs the product spectrum towards longer-chain products. Here, we show that acetate limitation induces hexanoate production via EO and RBO in a 1:2 stoichiometry. The ethanol uptake rate was ~ 6 × lower when electron acceptor (acetate or butyrate) limitation was imposed. Spirito et al.^34^ also observed a decreased conversion rate when feeding a chain-elongating reactor microbiome with mainly ethanol. This could explain why previous studies with shorter incubation times reported no metabolic activity in the absence of acetate^34,35^. The mechanism that underlies this change in rate remains elusive. Spirito et al.^34^ proposed a thermodynamic constraint on the rate due to increased hydrogen partial pressures, but in our experiments pH_2_ was low due to continuous sparging and we still observed low rates. Alternatively, the low concentrations of acetate and butyrate might hamper efficient transfer of the CoA-moiety from hexanoyl-CoA to a SCCA. This would limit the rate of the final step of RBO.

Furthermore, our data strongly suggest that ethanol conversion to hexanoate in the absence of an external electron acceptor is unstable and can trigger a transient change in reaction stoichiometry leading to electron acceptor production. A possible explanation is that in absence of butyrate the intracellular concentration of butyryl-CoA might become too low to render the condensation to 3-keto-hexanoyl-CoA feasible, triggering a metabolic shift towards transient butyrate production. This might explain that the presence of small amounts of butyrate (40–60 mCmol L^-1^) stabilized ethanol-only chain elongation. Intracellular metabolomics of chain-elongating cultures in different conditions could shed light on the influence of metabolite levels on the stoichiometries and rates of chain elongation^36^. Furthermore, using ^13^C-labelled ethanol might reveal whether butyrate is generated internally in the absence of acetate, or butyryl-CoA is directly consumed for the elongation to C_6_-intermediates^23^. However, a lack of commercially available standards for many of the intermediates of RBO impedes investigation of potential regulatory mechanisms in chain-elongating microbes.

**Figure 5:**
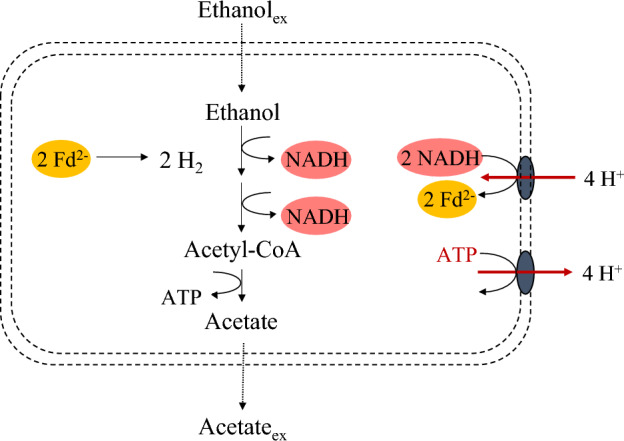
Ethanol oxidation and active export of protons via the ATPase. The reversal of the electron bifurcating Rnf complex drives the reduction of Fd, enabling hydrogen production. Rnf and ATPase stoichiometries based on Angenent et al.^17^.

A 1:2 stoichiometric coupling between EO and RBO was considered the extreme of ethanol-based chain elongation, but the organic acid production from ethanol observed here requires an EO:RBO ratio above 1:2. EO generates 2 mol of NADH (ΔE^0’^ (NADH/NAD^+^) = − 320 mV), which, in principle, does not provide enough reductive power for hydrogen (ΔE^0’^ (H_2_/2H^+^) = − 414 mV) formation. This is overcome by the electron bifurcating reaction in RBO, which lifts the electrons from NADH to ferredoxin (ΔE^0’^ (Fd^2-^/Fd) ~  − 500 mV). In the case of an EO:RBO ratio higher than 1:2, a different mechanism for NADH consumption and Fd^2-^ formation must have been employed to maintain the cellular redox balance. We suggest that reversal of the Rnf complex enabled the production of organic acids from ethanol. In this scenario, a chemiosmotic gradient over the membrane (Δψ) is built up by investing ATP conserved in EO and used to power the reduction of ferredoxin with NADH (Fig. 5). Bidirectionality of the Rnf complex has not been assessed in chain-elongating organisms, but has been demonstrated in other (an)aerobic organisms^32,37–40^.

The net ATP gain of EO with reversed Rnf activity is zero (Fig. 5). Coupling it to RBO can relieve this energetic constraint by reducing the ATP requirements for ferredoxin reduction by the Rnf complex. In the ethanol-only experiment, simultaneous production of either acetate/butyrate or butyrate/hexanoate was observed. Potential catabolic stoichiometries that could underly these physiologies are shown in Eqs. 6 and 7, respectively, and in Supplementary Figure S9.

6 $$3 Ethanol+{H}_{2}O\to Acetate+Butyrate+{2 H}^{+}+4 {H}_{2}$$

7 $$5 Ethanol\to Butyrate+ Hexanoate+2 {H}^{+}+4 {H}_{2}+{H}_{2}O$$

Verification of these stoichiometries with the experimental data is not trivial. The physiologies we observed occurred transiently and multiple stoichiometric couplings between EO and RBO render a redox balanced metabolism. Furthermore, the amounts of hydrogen evolved did not match the proposed stoichiometries exactly. This could also indicate that another electron sink was exploited, such as sulfate reduction^41^ or nitrogen fixation to NH_3_ via the nitrogenase enzyme^42,43^. Two subunits of the nitrogenase enzyme were more abundant under electron acceptor limitation (Supplementary Table S2). Physiological studies, for example under complete soluble nitrogen limitation or with nitrogenase-deficient mutants could elucidate the role of nitrogen fixation in chain-elongating organisms.

Lastly, the ethanol-only experiments strongly suggest that the same mechanistic change (i.e. Rnf reversal) does not consequently result in the same physiology^44^. Electron acceptor limitation proved to be stringent for the chain-elongating organisms and different metabolic tricks are effective for overcoming this stringency. It is remarkable that hexanoate production ceased in only one of the two replicates. The cease in hexanoate production could be explained if different enzymes catalyze the initiation or the entire second cycle of RBO. *C. kluyveri* possesses two non-identical sets of the same RBO enzymes and three different thiolase enzymes^43^ but their individual functionalities have not been thoroughly studied. To do so, knock-out studies with pure cultures of *C. kluyveri* are needed. This requires a genetic toolbox for *C. kluyveri,* which is not available to date and provides an interesting target for future work.

### Supplementary Information

Supplementary Information 1.

Supplementary Table S1.

Supplementary Table S2.

## Data Availability

Mass spectrometric raw data are available in the PRIDE partner repository under accession number PXD040972. Raw 16S rRNA gene amplicon sequencing data is available in the 4TU research database and can be found under the https://doi.org/10.4121/f74b503a-81d5-4187-b696-ba9c8133d2a3. All bioreactor data generated in this study is included in this published article and its supplementary information files.

## Human and animals rights

This article does not contain any studies with human participants or animals performed by any of the authors.
