# Author Correction: Physiological and stoichiometric characterization of ethanol-based chain elongation in the absence of short-chain carboxylic acids

**DOI:** 10.1038/s41598-024-62205-w

**Published:** 2024-05-23

**Authors:** Maximilienne Toetie Allaart, Bartholomeus B. Fox, Ingo H. M. S. Nettersheim, Martin Pabst, Diana Z. Sousa, Robbert Kleerebezem

**Affiliations:** 1 Department of Biotechnology, Delft University of Technology, Delft, The Netherlands; 2 Laboratory of Microbiology, Wageningen University & Research, Wageningen, The Netherlands

Correction to: *Scientific Reports* 10.1038/s41598-023-43682-x, published online 13 October 2023

The original version of this Article contained errors in the Discussion section, where reference 34 was incorrectly cited as reference 25. Consequently,

“Spirito et al.^25^ also observed a decreased conversion rate when feeding a chain-elongating reactor microbiome with mainly ethanol. This could explain why previous studies with shorter incubation times reported no metabolic activity in the absence of acetate^25,35^. The mechanism that underlies this change in rate remains elusive. Spirito et al.^25^ proposed a thermodynamic constraint on the rate due to increased hydrogen partial pressures, but in our experiments pH_2_ was low due to continuous sparging and we still observed low rates.”

now reads:

“Spirito et al.^34^ also observed a decreased conversion rate when feeding a chain-elongating reactor microbiome with mainly ethanol. This could explain why previous studies with shorter incubation times reported no metabolic activity in the absence of acetate^34,35^. The mechanism that underlies this change in rate remains elusive. Spirito et al.^34^ proposed a thermodynamic constraint on the rate due to increased hydrogen partial pressures, but in our experiments pH_2_ was low due to continuous sparging and we still observed low rates.”

The original Article has been corrected.

