# Supplementary material for: Physiological and stoichiometric characterization of ethanol-based chain elongation in the absence of short-chain carboxylic acids
Source: Sci Rep. 2023 Oct 13;13:17370. doi: 10.1038/s41598-023-43682-x (PMC10576071; doi:10.1038/s41598-023-43682-x)
Supplement: Supplementary file 1 — Supplementary Information 1. [file 41598_2023_43682_MOESM1_ESM.pdf]

Scientific Reports
Supplementary Materials to the article:

Physiological and stoichiometric characterization of ethanol-based chain elongation in the absence of short-chain carboxylic acids

Maximilienne Toetie Allaart[1], Bartholomeus B. Fox[1], Ingo H. M. S. Nettersheim[1], Martin Pabst[1], Diana Z. Sousa[2], Robbert Kleerebezem[1]

[1] Department of Biotechnology, Delft University of Technology, Delft, The Netherlands

[2] Laboratory of Microbiology, Wageningen University & Research, Wageningen, Netherlands

*** Correspondence:**
Maximilienne Allaart
M.T.Allaart@tudelft.nl

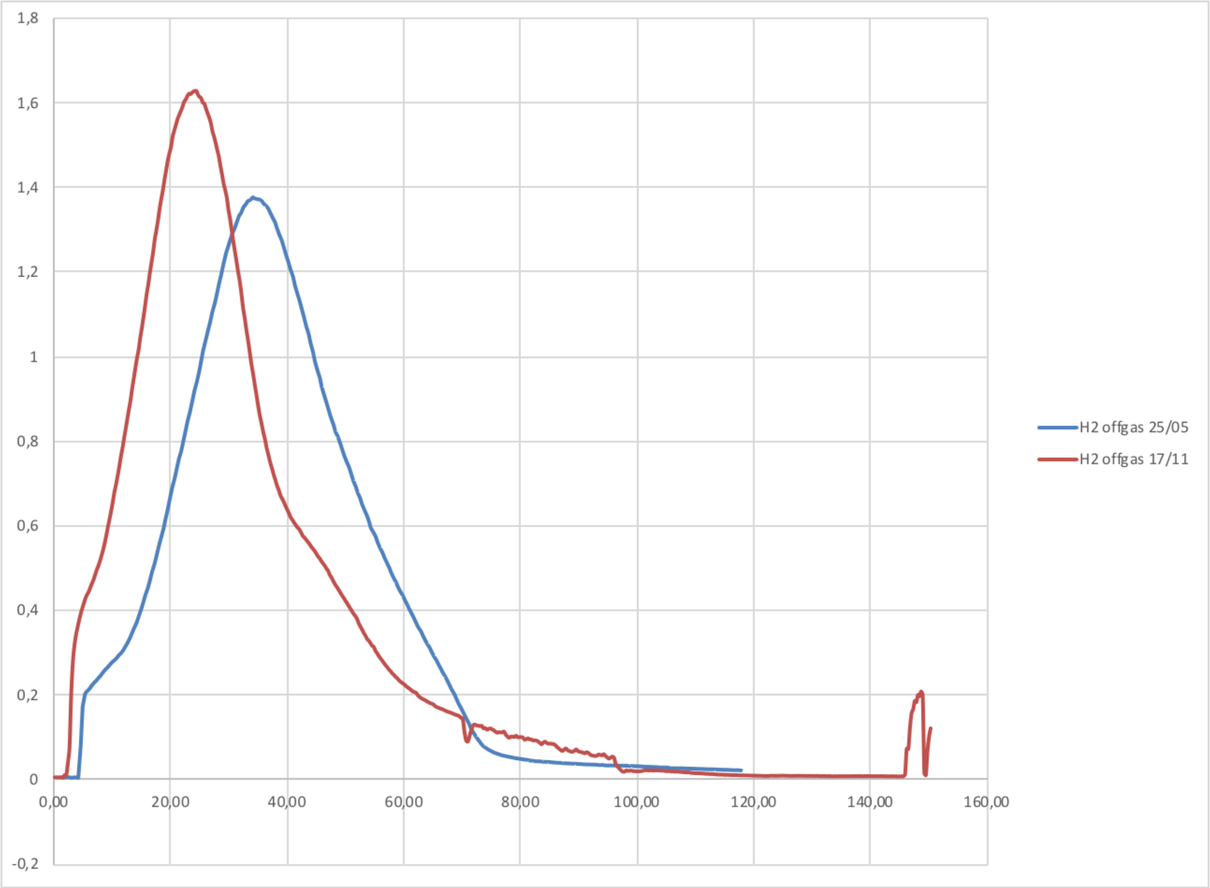

Figure S1. Raw hydrogen profiles of both biological replicates of the Ethanol + Acetate batch experiment (experiment 2). Differences in the peak time and height were attributed to slight differences in the activity of the inoculum. More importantly, the cumulative amounts of hydrogen produced, the total fermentation time and the shapes of the curves are similar. This confirms the replicability of the batch experiment.

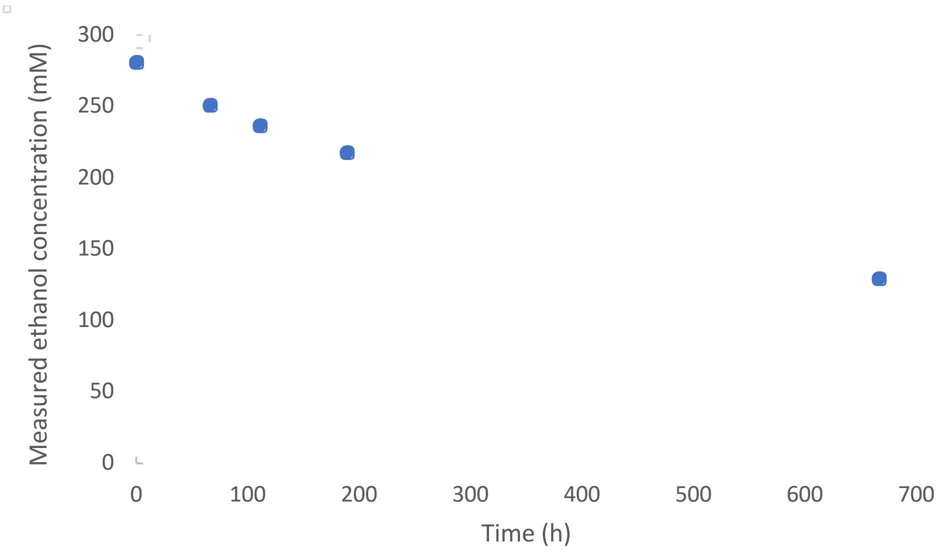

Figure S2. Abiotic bioreactor experiment for quantification of ethanol evaporation during long-term batch experiments. The experiment was carried out in the same bioreactor configuration as the batch experiments, with the same reactor volume, stirring and gas flow rates. Ethanol concentration was measured using HPLC.

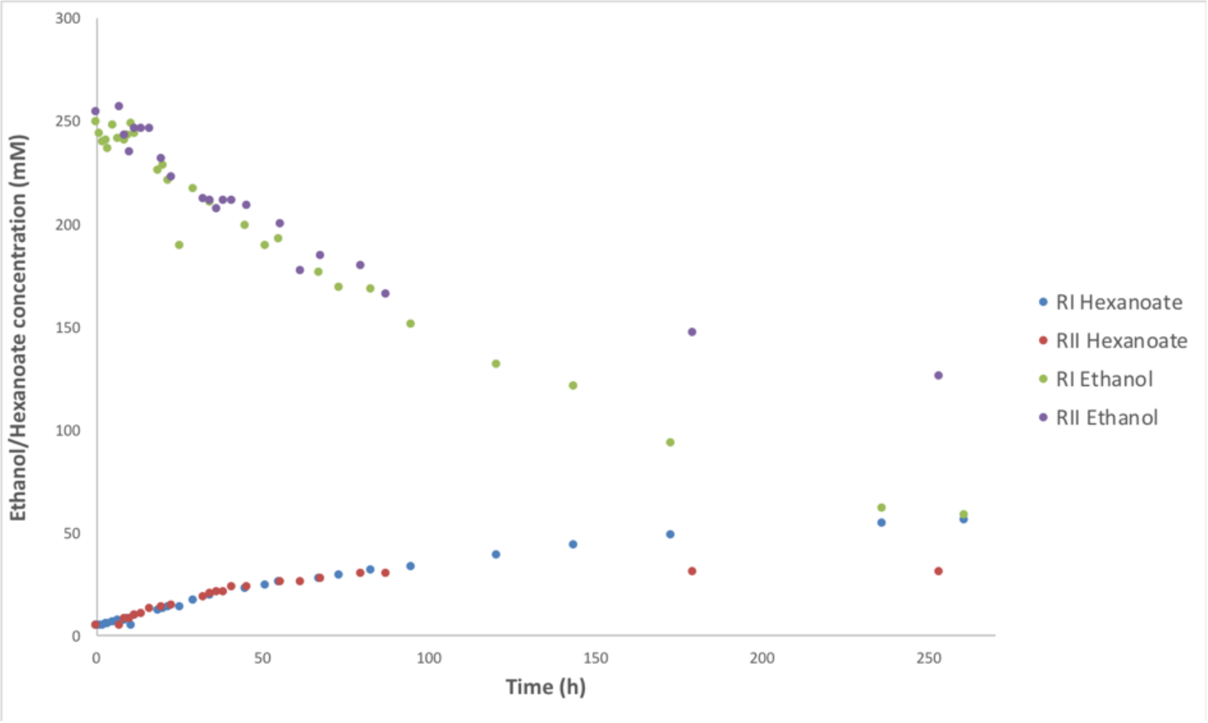

Figure S3. Ethanol and hexanoate profiles of both biological replicates of the Ethanol + Butyrate batch experiment (experiment 3). Highly replicable data was obtained up until approximately 100 hours of cultivation, after which metabolic activity ceased in replicate II. We hypothesize this was related to oxygen contamination.

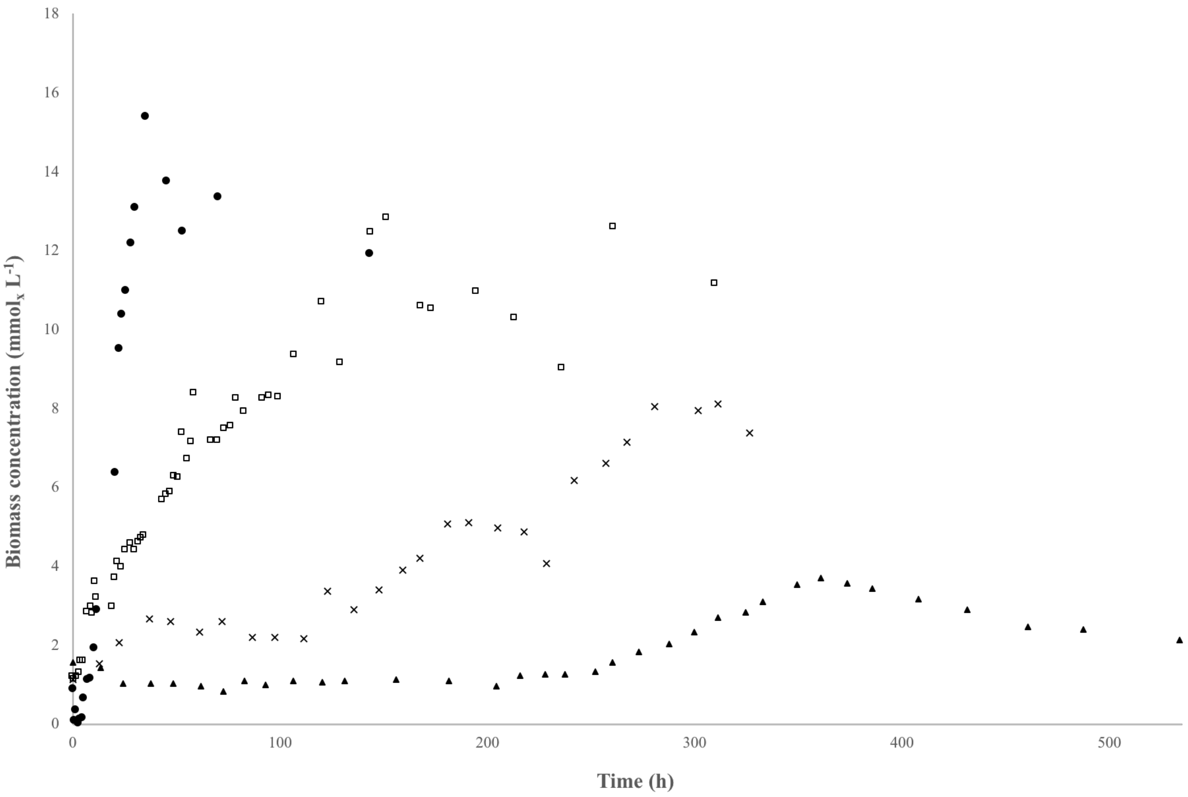

Figure S4. Biomass concentration in time during the different batch experiments.

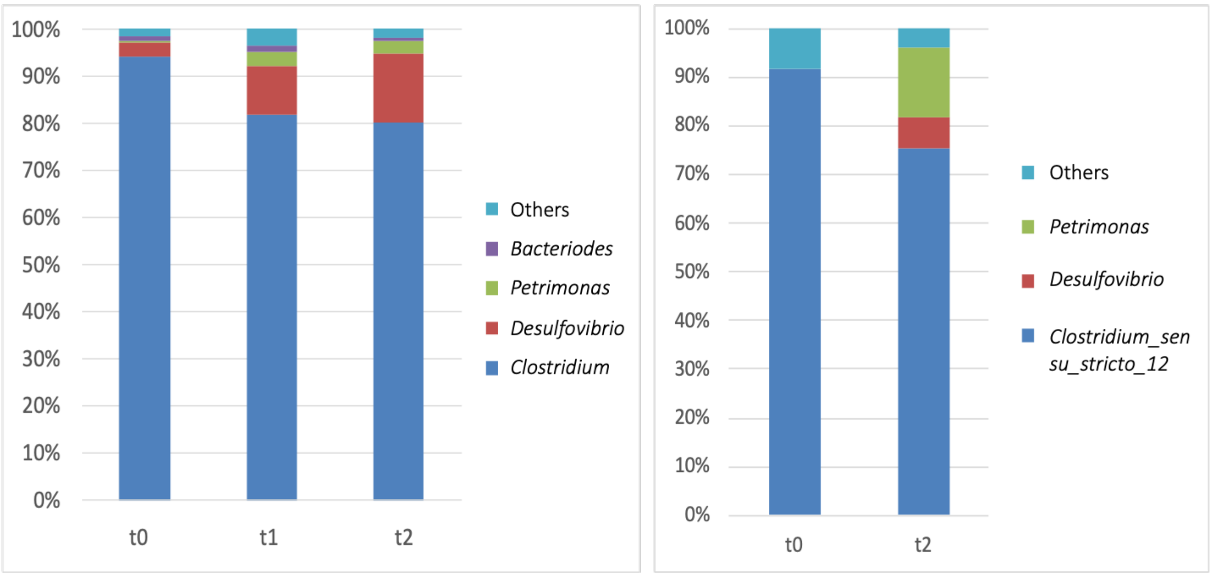

Figure S5. Estimation of the microbial composition using metaproteomics (left, protein biomass) and 16S rRNA gene amplicon sequencing (right, 16S rRNA) of the chain elongating microbial community in the ethanol-only batch experiment.

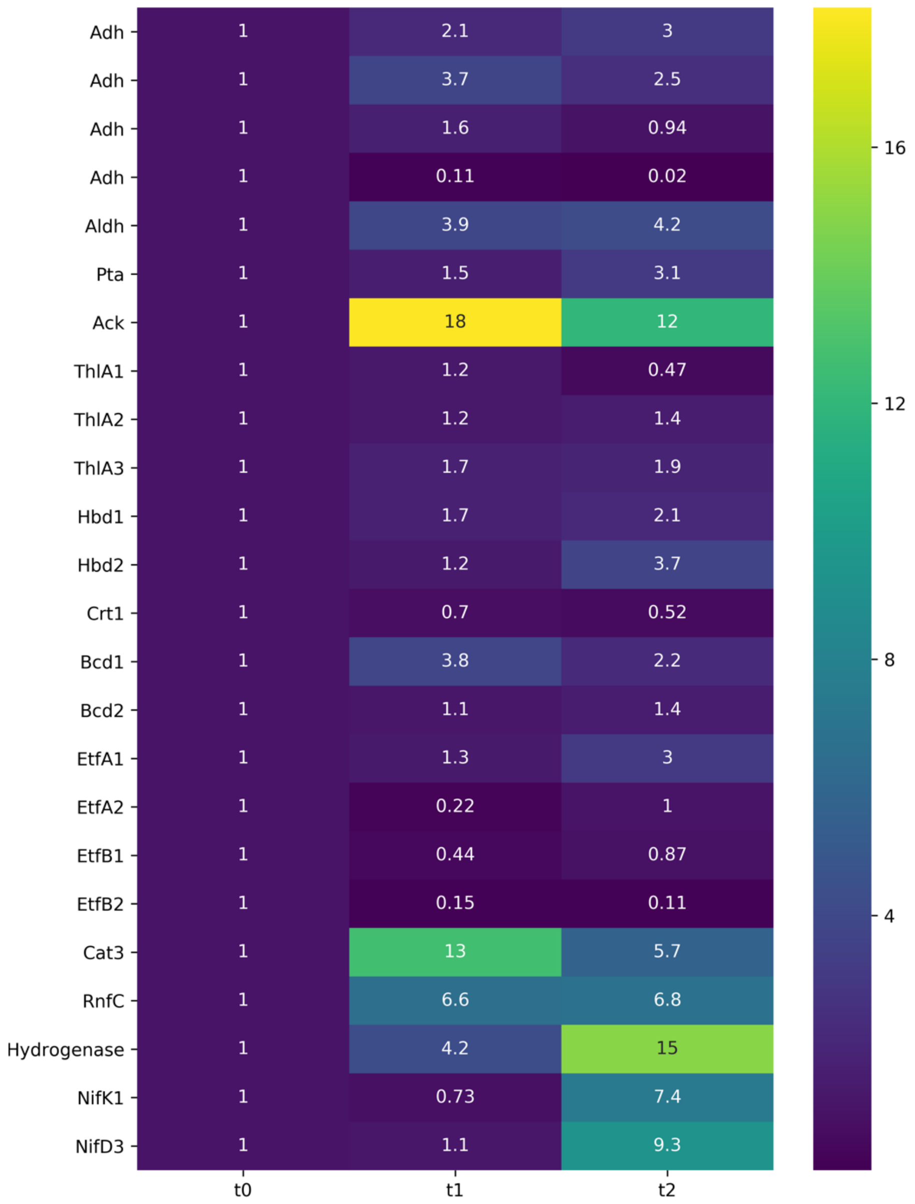

Figure S6. Heatmap of the log2 fold-change in protein levels of central carbon metabolism proteins and other specific proteins of interest. The log2 fold-change was calculated by taking the 2-base logarithm of the protein counts normalized to the number of protein counts in de inoculum. All changes were statistically significant ($p < 0.03$).

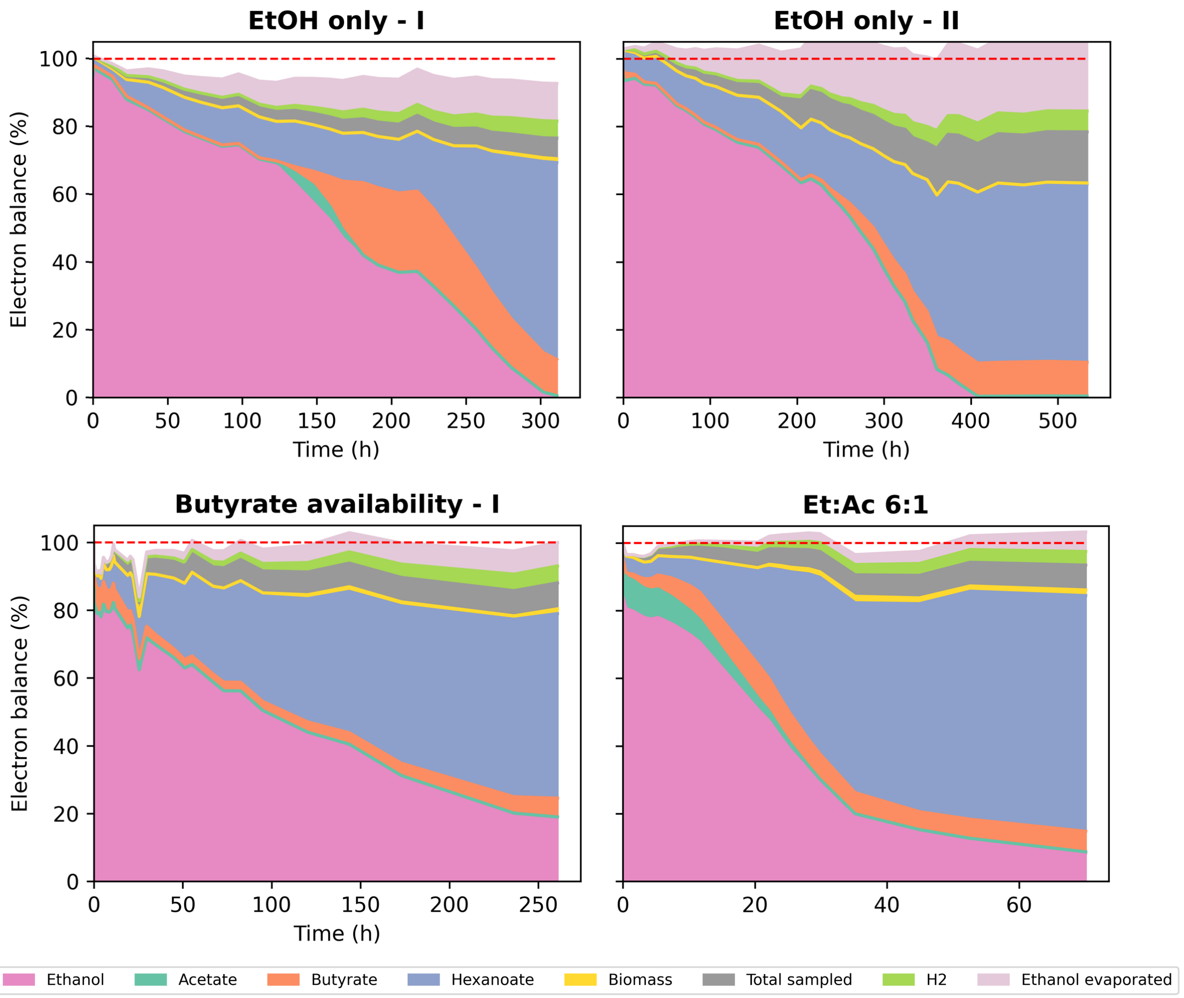

Figure S7. Electron distribution over time. The electron distribution was calculated and normalized considering the removal of substrates and products and the volume changes through sampling and acid/base addition. Ethanol evaporation was quantified using data from an abiotic ethanol evaporation test and the mass 15 signal data from the MS. The red dashed line is drawn at 100%, indicating a fully closed balance.

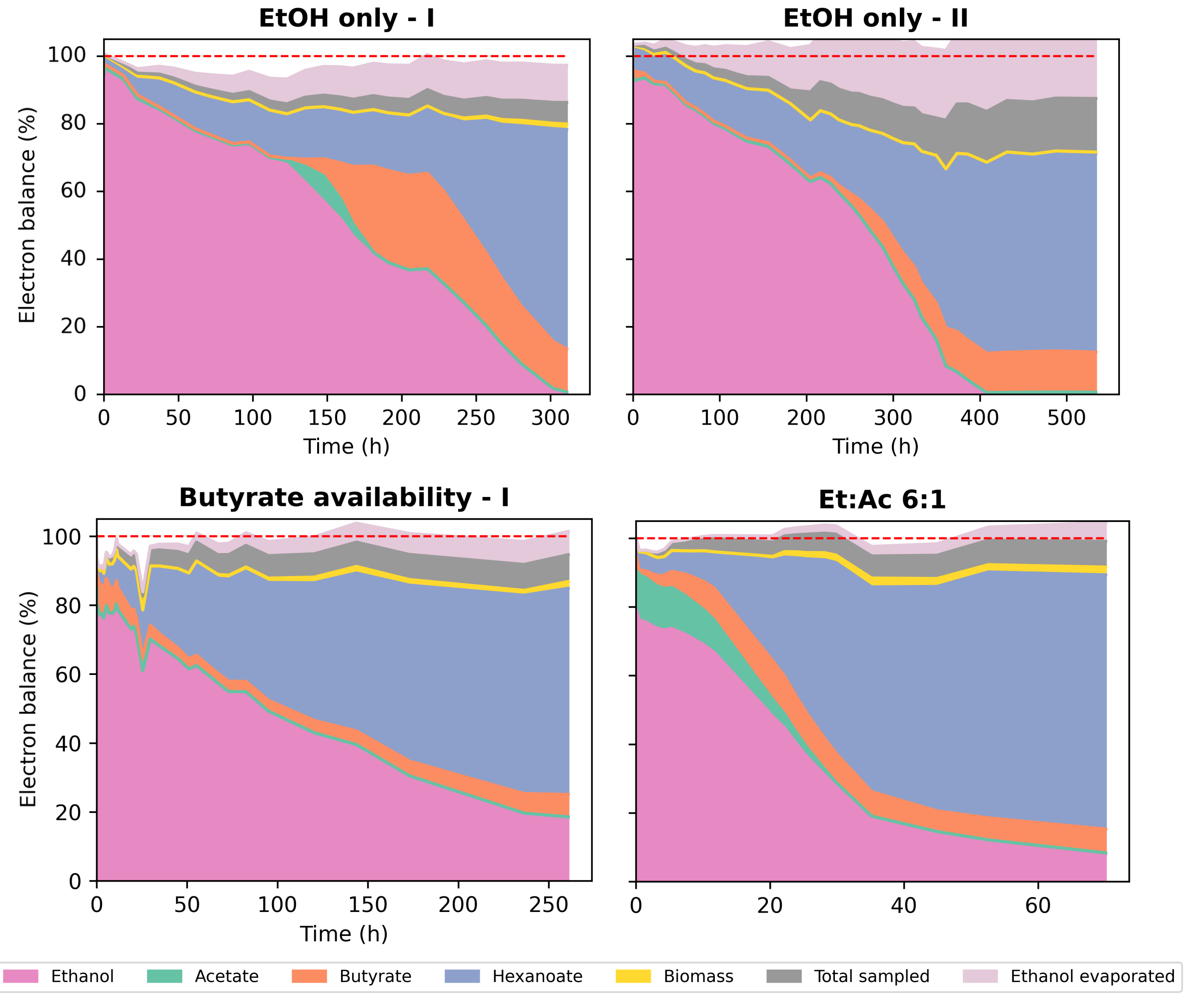

Figure S8. Carbon distribution over time. The carbon distribution was calculated and normalized considering the removal of substrates and products and the volume changes through sampling and acid/base addition. Ethanol evaporation was quantified using data from an abiotic ethanol evaporation test and the mass 15 signal data from the MS. The red dashed line is drawn at 100%, indicating a fully closed balance.

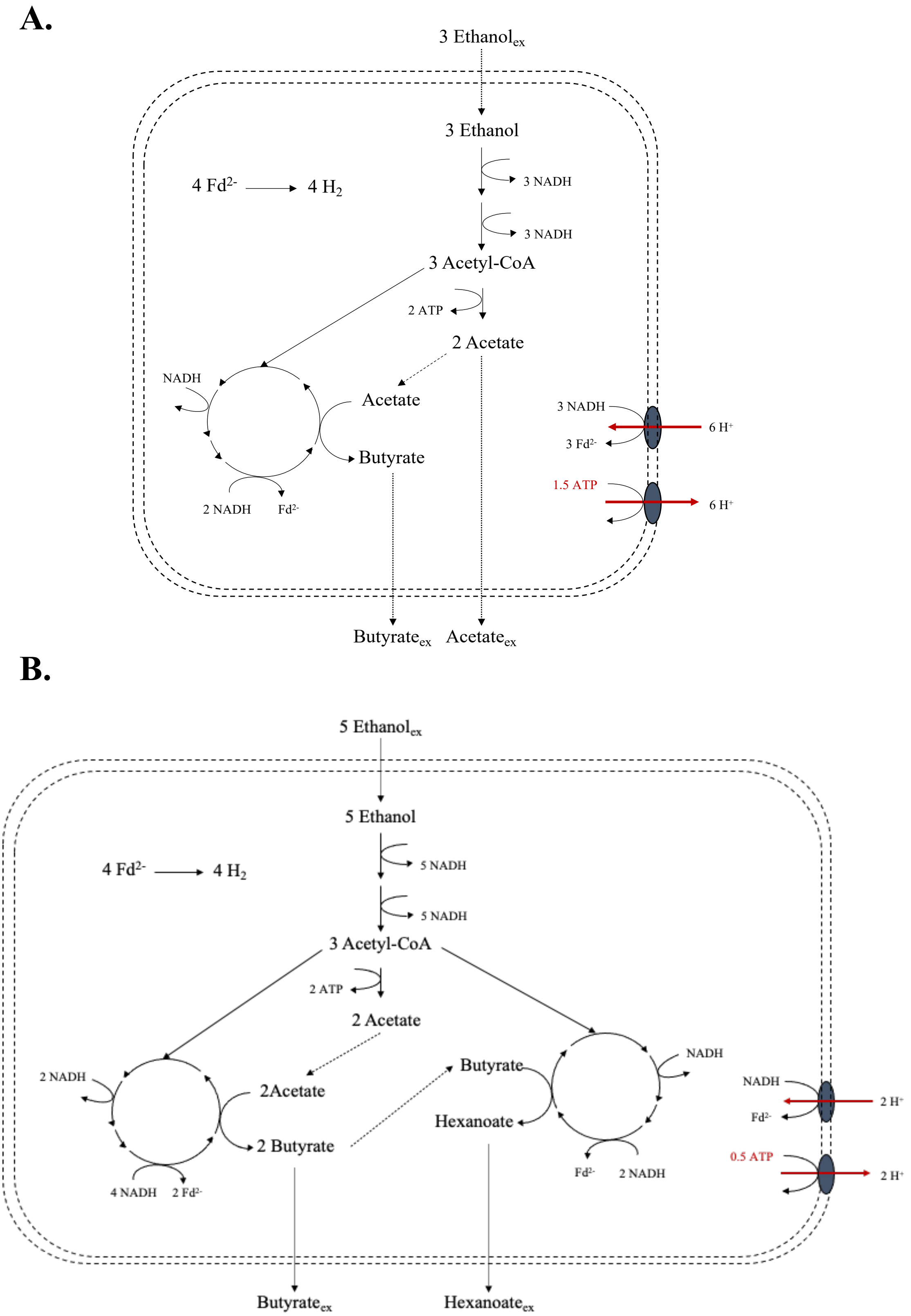

Figure S9. Stoichiometries for the simultaneous production of acetate, butyrate and hydrogen (A) or butyrate, hexanoate and hydrogen (B) and enabled by active export of protons via the ATPase and the reversal of the direction of the electron bifurcating Rnf complex. The reversal of the Rnf complex drives the reduction of Fd, enabling hydrogen production.

Table S1 – Excel file with raw data of batches (concentrations of products and substrates at all timepoints)

Table S2 – Excel file with differential proteomics analysis data – *C. kluyveri* database search
